# The psychometric properties of a shortened Dutch version of the consequences scale used in the Core Alcohol and Drug Survey

**DOI:** 10.1371/journal.pone.0187876

**Published:** 2017-12-07

**Authors:** Sara De Bruyn, Edwin Wouters, Koen Ponnet, Joris Van Damme, Guido Van Hal, the Task Force substance use in Flemish universities and colleges

**Affiliations:** 1 Department of Sociology, University of Antwerp, Antwerp, Belgium; 2 Department of Communication Studies, University of Antwerp, Antwerp, Belgium; 3 Department of Communication Studies, MICT-IMEC, Ghent University, Ghent, Belgium; 4 Association for Alcohol and other Drug problems, Brussels, Belgium; 5 Department of Epidemiology and Social Medicine, Medical Sociology and Health Policy, University of Antwerp, Antwerp, Belgium; Universita Cattolica del Sacro Cuore Sede di Roma, ITALY

## Abstract

**Background:**

Alcohol and drug misuse among college students has been studied extensively and has been clearly identified as a public health problem. Within more general populations alcohol misuse remains one of the leading causes of disease, disability and death worldwide. Conducting research on alcohol misuse requires valid and reliable instruments to measure its consequences. One scale that is often used is the consequences scale in the Core Alcohol and Drug Survey (CADS). However, psychometric studies on the CADS are rare and the ones that do exist report varying results. This article aims to address this imbalance by examining the psychometric properties of a Dutch version of the CADS in a large sample of Flemish university and college students.

**Methods:**

The analyses are based on data collected by the inter-university project ‘Head in the clouds’, measuring alcohol use among students. In total, 19,253 students participated (22.1% response rate). The CADS scale was measured using 19 consequences, and participants were asked how often they had experienced these on a 6-point scale. Firstly, the factor structure of the CADS was examined. Two models from literature were compared by performing confirmatory factor analyses (CFA) and were adapted if necessary. Secondly, we assessed the composite reliability as well as the convergent, discriminant and concurrent validity.

**Results:**

The two-factor model, identifying personal consequences (had a hangover; got nauseated or vomited; missed a class) and social consequences (got into an argument or fight; been criticized by someone I know; done something I later regretted; been hurt or injured) was indicated to be the best model, having both a good model fit and an acceptable composite reliability. In addition, construct validity was evaluated to be acceptable, with good discriminant validity, although the convergent validity of the factor measuring ‘social consequences’ could be improved. Concurrent validity was evaluated as good.

**Conclusions:**

In deciding which model best represents the data, it is crucial that not only the model fit is evaluated, but the importance of factor reliability and validity issues is also taken into account. The two-factor model, identifying personal consequences and social consequences, was concluded to be the best model. This shortened Dutch version of the CADS (CADS_D) is a useful tool to screen alcohol-related consequences among college students.

## 1. Introduction

The problematic use of alcohol and other drugs has been a worldwide concern for decades [1]. Globally, national and international policies and interventions have been installed aiming to tackle the harmful consequences of alcohol and drug misuse [1]. Action is especially needed with regard to alcohol misuse since it is the most prevalent psychoactive substance worldwide [2]. According to the World Health Organization, the problematic use of alcohol remains one of the five most important causes of disease, disability and death across the globe [1]. A staggering 5.9% of all deaths worldwide are caused by harmful alcohol use. Indeed, alcohol misuse has been indisputably identified as a public health problem [1]. This is especially true for young people, such as university and college students, as the transition from high school to university or college is often accompanied by high levels of substance use and more problematic alcohol use [3–8].

Alcohol use among students has been studied extensively in recent years and has received much media attention [1, 3, 4, 9]. A large-scale Flemish study indicated that 98% of university and college students have ever used alcohol and 93% of these students had drunk alcohol in the past 12 months. Half of all these students (49.7%) showed risk characteristics of problematic alcohol use [3]. Moreover, excessive consumption patterns such as binge drinking, have been reported as a common practice among young people [10], increasing the risk of experiencing alcohol-related consequences [10, 11]. Several studies have addressed the problematic consequences of students’ drinking behaviour, such as academic problems, injuries, assaults, driving under the influence and sexual assault [4, 12, 13], not only harming the student, but also other people around the student and within society as a whole. Given the immense burden that alcohol puts on society in terms of health, social and economic outcomes [1, 4, 14], it is vital that alcohol research is based on valid and reliable instruments to measure the consequences of alcohol misuse. In recent decades, considerable effort has been put into developing scales to measure the consequences of alcohol (and drug) use among young people [15–24]. However, reliability and validity testing of some of these instruments is lacking.

The Core Alcohol and Drug Survey (CADS) was developed in 1990 as a self-report instrument to assess the nature, scope and consequences of alcohol and other drug use amongst college students [24, 25]. Although numerous studies have used the consequences scale, a subscale of the survey [26–30], little attention has been paid to its psychometric properties. The initial developers presented this consequences scale as a unidimensional construct, without extensively investigating its factor structure, while another research study found that this scale had a two-factor structure that identified personal consequences (such as having a hangover) and consequences with others (such as getting into an argument or fight) [31]. Moreover, these studies were all performed in the US, creating a dearth of knowledge of the factor structure of the CADS in other contexts.

The primary aim of this research study is, therefore, to assess the psychometric properties of a Dutch version of the CADS in a large sample of 19,253 Flemish university and college students. As alcohol is currently the most prevalent psychoactive substance worldwide, we focus especially on assessing the scale with regard to alcohol consequences. We examined the factor structure of the Dutch CADS by comparing the one- and two-factor model as presented in the literature by using confirmatory factor analysis and adapting the models if necessary [32, 33]. In addition, we tested for composite reliability and both construct (i.e., convergent and discriminant) and criterion-related (i.e., concurrent) validity to verify the consistency as well as the accuracy of the factors.

## 2. Materials and methods

### 2.1 Procedure and participants

The analyses are based on data collected by the inter-university project ‘Head in the clouds’ [3]. A cross-sectional survey was sent to students of the eleven universities and colleges in Flanders (Belgium) who were willing to participate. Students were invited by email and other methods (e.g., student magazine) to participate anonymously to an online survey. They had four to six weeks to participate in the period February to April 2013 and no reminder was sent [3]. Students could voluntarily decide whether or not to participate by actively clicking on the link in the email which would lead them to the online survey. The introduction clearly stated that the data would be anonymous. To increase response rate, some incentives (e.g., the chance to win a number of prizes, including an iPad) were offered to the participating students and only if they agreed to provide an email address. Five colleges were excluded from the sample because they had a very low response rate (< 3.5%). This resulted in a final sample of 19,253 college and university students (22.1% response rate). The study was performed according to the ethical standards of the American Psychological Association and was approved by the Ethics Committee of Ghent University Hospital (EC UZG 2013/065).

Of the 19,253 participants, 35.7% (n = 6,867) were male and 64.3% (n = 12,386) were female. Mean age was 21.12 years (SD = 3.251). Table 1 provides an overview of sex and age distributions among participating institutions. We also performed bivariate analyses (ANOVAs) with age as the dependent variable and sex as well as institution as the group variable to verify any significant differences in participants’ age between men and women and between institutions. Results indicated that age significantly differs between institutions (*F*(5) = 49.733, *p* < 0.000). With regard to sex, however, no significant difference was found between the age of male and female participants (*F*(1) = 0.117, *p* = 0.732).

**Table 1:** Sex and age distributions among participating institutions.

	Sex (% (n))		Age (mean (SD))
	Male	Female	
University of Antwerp (n = 1,897)	32.4 (614)	67.6 (1,283)	22.05 (4.273)
University of Ghent (n = 7,181)	37.8 (2,711)	62.2 (4,470)	21.07 (2.182)
University of Leuven (n = 5,189)	33.0 (1,713)	67.0 (3,476)	20.80 (3.046)
KdG College (n = 2,248)	32.3 (725)	67.7 (1,523)	21.20 (3.825)
KHLimburg College (n = 2,087)	32.1 (669)	67.9 (1,418)	20.93 (3.425)
Group T College (n = 651)	66.8 (435)	33.2 (216)	21.84 (2.487)
Total (n = 19,253)	35.7 (6,867)	64.3 (12,386)	21.12 (3.251)

### 2.2 Measures

*Negative consequences of alcohol use* were measured using the CADS [34]. Participants were asked how often they have experienced a list of 19 consequences (e.g., got into an argument or fight) as a consequence of their drinking or drug use during the last year. The internal consistency of the items was reported to be high with a Cronbach’s Alpha of 0.90 [24]. The CADS was translated into Dutch by two independent translators. Both translations were almost similar. Any differences that do existed were discussed in the working group responsible for the questionnaire. Moreover, five students pre-tested the usability and comprehensibility of the questionnaire. The answer categories of the CADS were ‘none’, ‘one’, ‘two’, ‘three to five’, ‘six to nine’ and ‘10 or more times’. Frequencies were coded using mid-points of the categories, respectively 0, 1, 2, 4, 7.5 and 11.25 times for the upper category (10 times plus half range to midpoint of adjacent category) [35]. The complete list of consequences is presented in S1 Table.

*The Alcohol Use Disorder Identification Test (AUDIT)* was developed by the World Health Organization (WHO) and measures problematic alcohol use with 10 items [36]. The scale has proven to be useful and reliable in measuring problematic alcohol use among students [37, 38]. The AUDIT was officially translated into Dutch with the approval of the WHO [39] and has proven to be a reliable screening instrument [40]. In this study, we used the shortened version, the AUDIT-c, which has proven to be an equally good or even better indicator for measuring problematic alcohol use [41–43]. The AUDIT-c consists of three questions, measured on a 5-point scale: ‘How often do you drink alcohol (in general)’; ‘if you drink, how many glasses do you usually drink per day’; ‘how often does it happen that you drink six glasses or more in one single occasion’. The reliability of the AUDIT-c in the present study was good (α = 0.795).

*Binge drinking w*as measured by asking students to indicate how often they drank four glasses or more (for women) or six glasses or more (for men) during a time span of two hours. One glass refers to a standard glass of alcohol containing 10 g or 12.7 ml pure alcohol. This amount corresponds to approximately 1 glass of beer (25 cl), wine (10 cl), non-distilled beverage such as sherry (5 cl), or spirits (3.5 cl) [44]. Response options ranged from 1 = never, 2 = less than monthly, 3 = monthly, 4 = weekly, to 5 = daily or almost daily. The time-frame used to measure binge drinking was within the previous year.

### 2.3 Analytic strategy

Data were analyzed using IBM SPSS Statistics 22 and IBM SPSS Amos 22. We only included those participants who reported drinking alcohol within the past 12 months (n = 17,756) in the analyses. Firstly, we performed descriptive analyses to describe drinking characteristics and the related alcohol consequences in our sample. Next, we examined the factor structure of the CADS by performing confirmatory factor analyses. The analyses are a mix of the alternative models approach and a model generating approach, as defined by Jöreskog [33], in which we compare two models as presented in the literature and modify them with the goal of finding a model that fits the data well and has a theoretically meaningful interpretation. We started with the one-factor model as described by Presley (i.e., Model 1a) [24] and the two-factor model indicated by Martens et al. (i.e., Model 2a) [31]. These initial models were adapted and compared, based on model fit and their composite reliability. Martens et al. (2005) made several decisions in their analyses to improve model fit. First of all they excluded all the items experienced by 5% or less of the participants. In addition, they excluded items 11 (had a memory loss) and 12 (done something I later regretted) as they loaded high on both factors. We employed a similar strategy for our data.

We used several goodness-of-fit indices to measure model fit. The classic goodness-of-fit index is χ^2^. However, it is well known that χ^2^ is almost always significant in the case of large sample sizes [45]. We therefore also reported the Root Mean Square Error of Approximation (RMSEA), Standardized Root Mean Square Residual (SRMR), Comparative Fit Index (CFI) and Tucker-Lewis Index (TLI). We also reported the Akaike Information Criterion (AIC), as this index allows a comparison between non-nested models. The following (strict) cutoff criteria were used to evaluate model fit: SRMR < 0.08 [46]; RMSEA < 0.08 = adequate fit; < 0.05 = good model fit [47]; CFI and TLI > 0.95 [46]; factor loading (FL) > 0.50 [45].

Since the CADS is not normally distributed (0 is very frequently answered), we used the ADF estimator in AMOS [45]. Item 1 is the reference item in the one-factor model. In the two-factor model, item 1 is the reference item for the ‘personal consequences’ factor and item 19 is the reference item for the factor ‘social consequences’. We used Jöreskog Rho = (Sum(FL))^2^ / ((Sum (FL))^2^ + Sum (1-FL^2^)) to evaluate the composite reliability of every model [48].

We also tested the validity of the best fitting model. As indicated by the International Test Commission, we provided evidence on both construct validity as well as criterion-related validity [49]. First of all, construct validity was measured by both convergent and discriminant validity. As Brown (2006) describes, “*convergent validity is indicated by evidence that different indicators of theoretically similar or overlapping constructs are strongly interrelated”* [45]. In other words, all items of one construct need to be interrelated with factor loadings above 0.50 (or even better above 0.70). A more strict evaluating tool of convergent validity is measuring average variance extracted (AVE = (Sum of FL^2^)/(Sum of FL^2^+ Sum (1-FL^2^)). Strictly speaking the AVE needs to be higher than 0.50 [48]. Discriminant validity *“is indicated by results showing that indicators of theoretically distinct constructs are not highly intercorrelated”* [45]. In other words, we do not want items of one construct loading onto another construct, or items of different constructs correlating with each other. The covariance of factors needs to be lower than 0.80–0.85 [45]. Secondly, we also addressed the concurrent validity by replicating a well-known correlation with two external variables (binge drinking and AUDIT-c). Missing items were deleted using listwise deletion.

## 3. Results

### 3.1 Descriptive results

Table 2 provides the sample responses on binge drinking and on the AUDIT-c. Table 3 gives an overview of the item score distribution of the CADS.

**Table 2:** Drinking characteristics of the sample.

Binge drinking (% (n))	Never	38.4 (6612)
	Less than monthly	37.7 (6487)
	Monthly	15.5 (2665)
	Weekly	8.1 (1387)
	(Almost) daily	0.3 (48)
AUDIT-c (% (n))		
AUDIT1 - ‘How often do you drink alcohol (in general)’?	Never	1.4 (242)
	Monthly or less	23.5 (4071)
	Once a week or less	36.9 (6394)
	2 to 3 times a week	31.0 (5370)
	4 times a week	7.1 (1237)
AUDIT2 –‘If you drink, how many glasses do you usually drink per day’?	1 or 2	42.5 (7286)
	3 or 4	33.1 (5666)
	5 or 6	14.6 (2507)
	7 to 9	6.6 (1128)
	10 times or more	3.2 (546)
AUDIT3 - ‘How often does it happen that you drink six glasses or more in one single occasion’?	Never	25.9 (4441)
	Less than monthly	34.6 (5947)
	Monthly	21.4 (3682)
	Weekly	17.2 (2955)
	(Almost) daily	0.9 (149)

**Table 3:** Item score distribution of the CADS.

CADS ITEMS	Percentage of answers in each frequency category							
	Never	Once	Twice	3–5 times	6–9 times	10 times or more	Mean	Standard Deviation
1	31.2	15.1	12.4	17.6	9.5	14.3	3.41	3.89
2	86.5	6.8	3.4	2.4	0.6	0.3	0.31	1.10
3	95.6	3.2	0.8	0.3	0.0	0.0	0.07	0.44
4	95.0	3.2	1.0	0.6	0.1	0.1	0.09	0.56
5	87.1	8.0	2.9	1.5	0.2	0.2	0.24	0.87
6	41.0	26.2	15.2	11.9	3.3	2.4	1.55	2.28
7	91.4	4.6	1.6	1.2	0.5	0.7	0.24	1.19
8	54.2	12.2	10.0	11.4	4.9	7.4	1.97	3.24
9	77.0	11.2	5.9	3.7	1.0	1.2	0.59	1.63
10	94.5	2.5	1.4	0.8	0.3	0.5	0.17	1.00
11	69.5	12.3	7.7	5.8	2.3	2.4	0.95	2.18
12	67.3	17.0	8.6	5.1	1.2	0.8	0.73	1.58
13	99.7	0.2	0.0	0.0	0.0	0.0	0.01	0.24
14	99.1	0.7	0.1	0.0	0.0	0.0	0.02	0.28
15	99.8	0.1	0.1	0.0	0.0	0.0	0.01	0.27
16	97.1	1.4	0.7	0.5	0.1	0.2	0.08	0.63
17	96.7	1.7	0.6	0.5	0.2	0.3	0.10	0.79
18	99.5	0.3	0.1	0.1	0.0	0.0	0.01	0.27
19	87.2	7.7	3.2	1.3	0.3	0.2	0.24	0.90

### 3.2 Fit of the one-factor models

#### Model 1a

We started by testing the one-factor structure of the CADS, containing all of the 19 items. As shown in Tables 4 and 5, the fit of model 1a was bad, except for the RMSEA. 14 of the 19 factor loadings were below 0.50, and the factor loadings of items 13, 14 and 18 were not significant on a *p* < 0.001 level. Composite reliability was good with rho = 0.710.

**Table 4:** Factor loadings, significance, and composite reliability of the models.

	Model 1a: one-factor model 19 items	Model 1b: one-factor model 11 items	Model 1c: one-factor model 5 items	Model 2a: two-factor model (pers.: 1, 6, 7, 8; soc.: 2, 3, 4, 5, 9, 10, 19)		Model 2b: two-factor model (pers: 1, 6, 8; soc: 5, 9, 19)		Model 2c: two-factor model (pers: 1, 6, 8; soc: 5, 9, 12, 19)	
CADS items				Personal cons.	Social cons.	Personal cons.	Social cons.	Personal cons.	Social cons.
**1**	0.840 (ref. cat.)	0.837 (ref. cat.)	0.827 (ref. cat.)	0.835 (ref. cat.)		0.830 (ref. cat.)		0.821 (ref. cat.)	
**2**	0.354***	0.352***			0.435***				
**3**	0.245***				0.333***				
**4**	0.265***				0.365***				
**5**	0.392***	0.396***			0.463***		0.501***		0.491***
**6**	0.669***	0.670***	0.680***	0.678***		0.681***		0.685***	
**7**	0.206***	0.204***		0.203***					
**8**	0.670***	0.679***	0.672***	0.683***		0.671***		0.685***	
**9**	0.404***	0.405***			0.510***		0.552***		0.548***
**10**	0.236***	0.225***			0.300***				
**11**	0.607***	0.609***	0.624***						
**12**	0.578***	0.582***	0.588***						0.729***
**13**	0.032*								
**14**	0.046**								
**15**	0.019***								
**16**	0.154***								
**17**	0.082***								
**18**	0.024								
**19**	0.408***	0.419***			0.489 (ref. cat.)		0.525 (ref. cat.)		0.514 (ref. cat.)
Composite reliability									
**Jöreskog Rho**	0.710	0.784	0.812	0.711	0.592	0.773	0.534	0.776	0.662

Significance levels

* *p* < 0.05

** *p* < 0.01

*** *p* < 0.001

Ref. cat. refers to the reference category as explained in section 2.3 Analytic strategy

**Table 5:** Goodness-of-fit indices of the 6 models.

Goodness-of-fit indices	Model 1a	Model 1b	Model 1c	Model 2a	Model 2b	Model 2c
**χ^2^**	798.126	594.519	234.790	407.528	174.137	202.125
**df**	152	44	5	43	8	13
***p***	0	0	0	0	0	0
**RMSEA**	0.016	0.028	0.053	0.023	0.036	0.030
**SRMR**	0.175	0.097	0.040	0.088	0.034	0.033
**CFI**	0.868	0.882	0.948	0.916	0.960	0.956
**TLI**	0.851	0.852	0.897	0.893	0.925	0.929
**AIC**	874.126	638.519	254.790	453.528	200.137	232.125

*p* = significance level

#### Model 1b

We excluded certain items as they were rarely endorsed (i.e., 5% or less) by the participants [31]. This resulted in an exclusion of 8 items, namely items 3, 4, 13, 14, 15, 16, 17 and 18. The 11-item scale was tested as a one-factor model. As shown in Tables 4 and 5, the model fit was not good. RMSEA indicated a good model fit, but the other fit indices clearly did not. Some factor loadings were still low [loadings ranging from 0.204 (item 7) to 0.837 (item 1)], although all loadings were significant. Composite reliability was adequate with rho = 0.784.

#### Model 1c

Because model 1b did not have an acceptable fit, we eliminated one by one all the items with a low factor loading (standardized loading < 0.50) from our analyses. After each elimination, we evaluated the model fit, resulting in a one-factor structure containing 5 items (1, 6, 8, 11, 12). Standardized factor loadings were higher than 0.50 and all were highly significant (*p* < 0.001). This model was seen to be the ‘best’ model of all the one-factor models. As shown in Table 5, the model had an acceptable model fit, although CFI, and especially TLI could be improved. Composite reliability was calculated to be 0.812.

**Fig 1:**
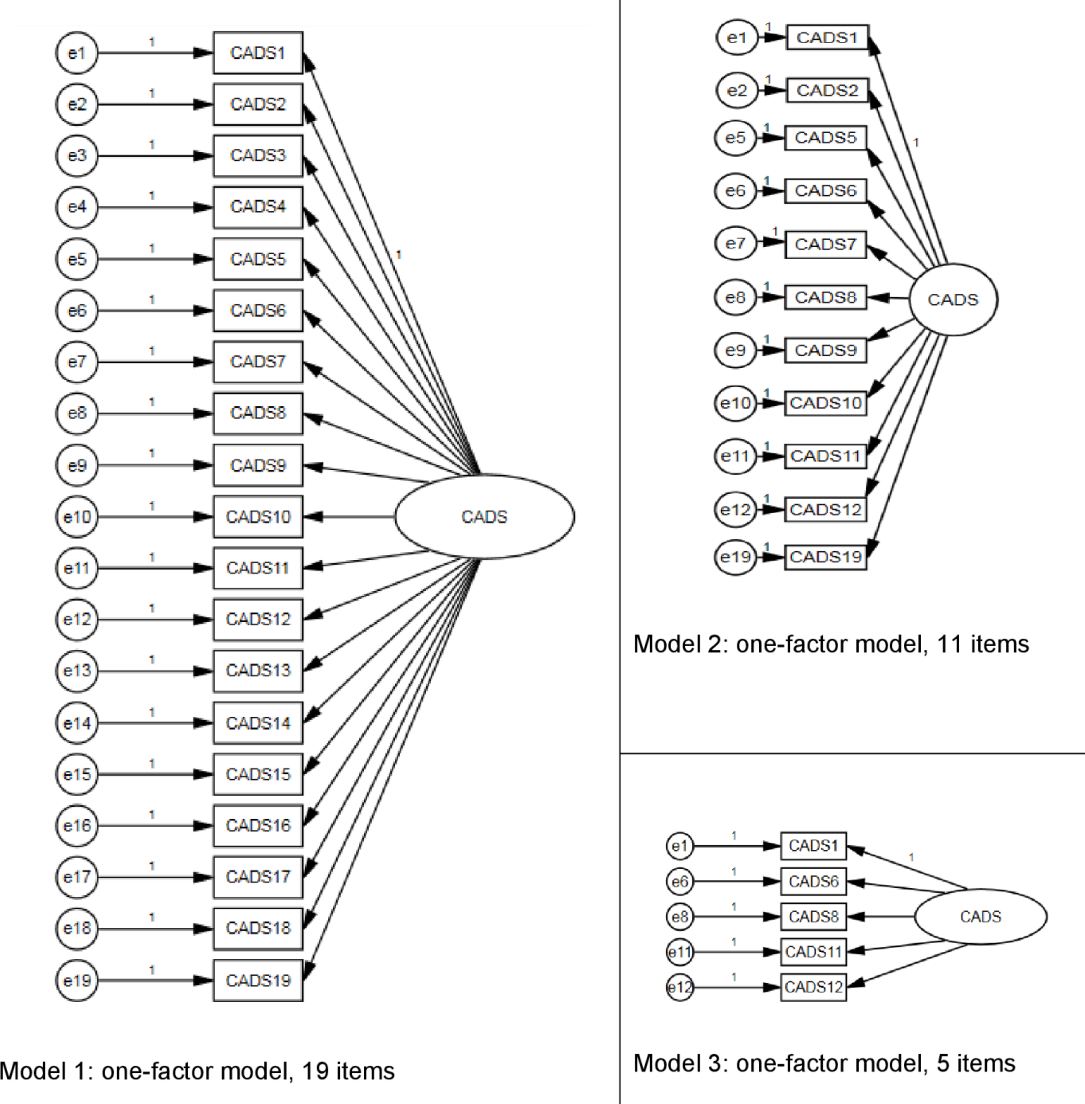
Overview of the three one-factor models.

Fig 1 presents the one-factor models.

### 3.3 Fit of the two-factor models

#### Model 2a

We first tested the two-factor model as described in Martens et al. (2005), identifying personal consequences (items 1, 6, 7 and 8) and consequences with others (items 2, 3, 4, 5, 9, 10, 19) which we further refer to as social consequences. We used CFA with correlated factors (similar to an oblique rotation) to test this model. The results are presented in Tables 4 and 5. All loadings were significant, but not all of them were higher than 0.50. Moreover, the model fit was not good (χ^2^ = 407.528; RMSEA = 0.023; SRMR = 0.088; CFI = 0.916; TLI = 0.893; AIC = 453.528). Correlation of the two factors was 0.82. Composite reliability was good for factor 1 (0.711), but not for factor 2 (0.592).

However, as our dataset is different from that of Martens et al. (2005), we extrapolated the decisions they made (cfr. 2.3 Analytic strategy) to our dataset and tested two additional models. As items 3 and 4 were experienced by less than 5% of the participants, these items were also excluded in our analyses (Model 2b). Since we did not know whether items 11 and 12 would load high on both factors, we included them in model testing (Model 2c). In the two models we eliminated items if necessary.

#### Model 2b

When testing the initial Model 2b, we concluded that the model fit was similar to Model 2a. Factor loadings were significant, but some were really low (< 0.50). As a consequence, these items were deleted one by one and model fit was evaluated each time. This process of testing and evaluating the fit resulted in the following model: Personal consequences (items 1, 6, 8) and Social consequences (items 5, 9, 19). The results of this model are shown in Table 4. All factor loadings were significant and higher than 0.50. The model fit was good, as can be seen in Table 5 (χ^2^ = 174.137; RMSEA = 0.036; SRMR = 0.034; CFI = 0.960; TLI = 0.925; AIC = 200.137). Correlation of the two factors was 0.76. Composite reliability was good for factor 1 (0.773), but not for factor 2 (0.534).

#### Model 2c

Model 2c was based on Model 2b, but included items 11 and 12 as well. It was clear that item 11 ‘had a memory loss’ belonged to the factor of personal consequences. For item 12 ‘done something I later regretted’, however, it was somewhat unclear whether it is a consequence that only relates to the drinker or to other people as well. We therefore performed two CFA’s: one where item 12 was part of factor 1 and another where it belonged to factor 2. As the second CFA gave a better fit (AIC of 409.557 compared to 385.866), we included item 12 in the factor of social consequences.

However, since item 11 had a high cross loading (similar to Martens et al. (2005)) with the factor social consequences, we still excluded item 11 from the model. This resulted in a major improvement of the model fit. This process of testing and evaluating fit resulted in the following model: Personal consequences (items 1, 6, 8) and Social consequences (items 5, 9, 12, 19). The results of this model are shown in Tables 4 and 5. Factor loadings were all significant and model fit was good (χ^2^ = 202.125; RMSEA = 0.030; SRMR = 0.033; CFI = 0.956; TLI = 0.929; AIC = 232.125). Correlation between the two factors was 0.78 and composite reliability for the two factors was 0.776 and 0.662.

**Fig 2:**
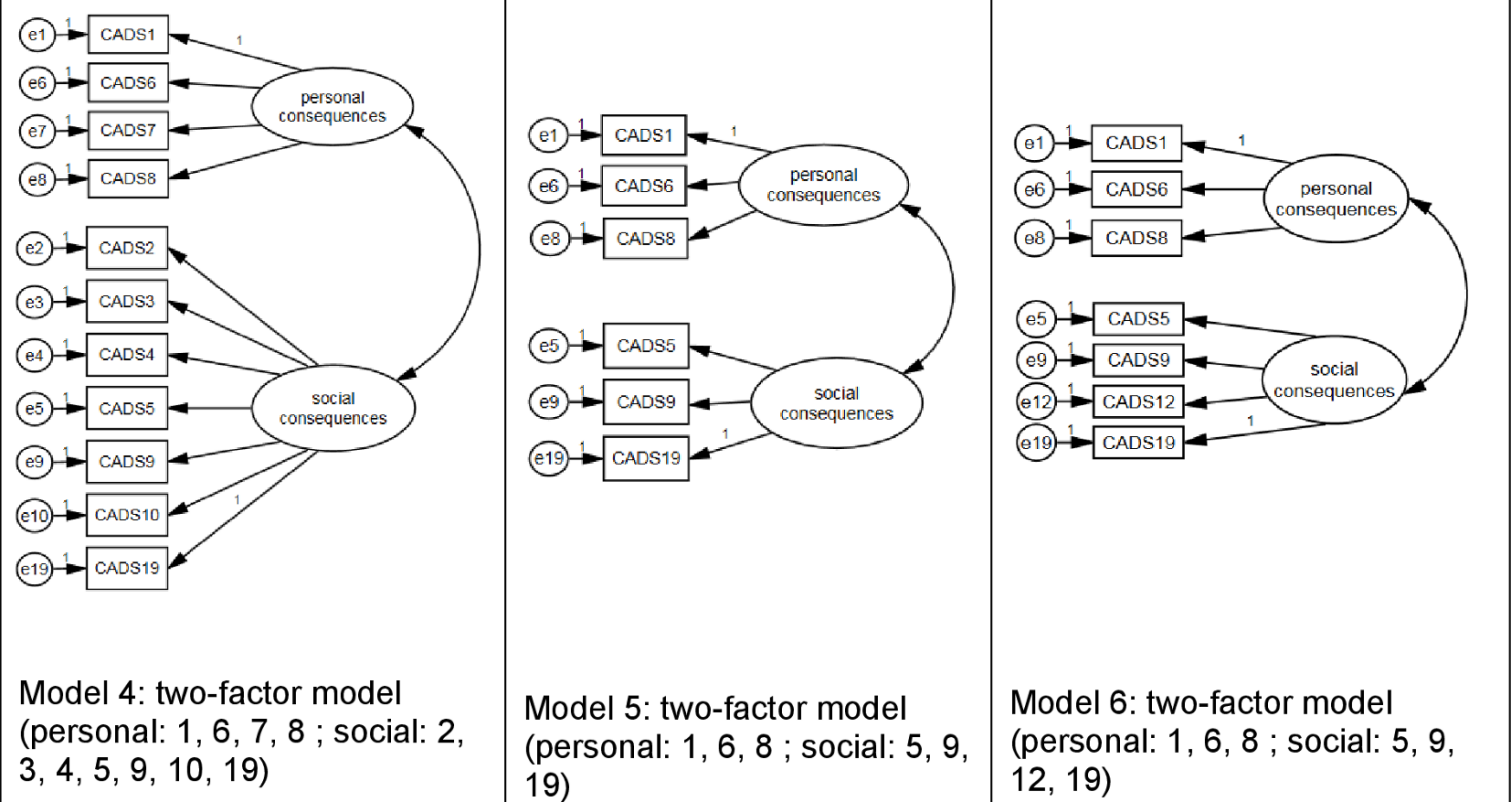
Overview of the three two-factor models.

Fig 2 presents the two-factor models.

### 3.4 Conclusion ‘best’ model

We performed CFA’s on both one- and two-factor structures of the CADS, starting from two models in the literature [24, 31], and adapting them based on the (significance of) factor loadings, modification indices and goodness-of-fit indices. As shown in Table 5, Model 2b has the lowest AIC and thus the best model fit, closely followed by Model 2c. However, the factor ‘social consequences’ of Model 2b has a low composite reliability, which is much better in Model 2c. In deciding which model best represents the data, it is crucial that not only model fit is evaluated, but also composite reliability is taken into account. Based on this, it can be concluded that Model 2c is the best fitted model in understanding the consequences of alcohol misuse, as it has both a good model fit and an acceptable composite reliability. In the next step, we test the construct and concurrent validity of this model.

### 3.5 Construct and concurrent validity

We evaluated the validity of Model 2c and we focused on both construct and concurrent validity.

#### 3.5.1 Construct validity

Construct validity was measured by both convergent and discriminant validity. As described in the analytic strategy (section 2.3), all items of a construct need to be highly interrelated (factor loadings > 0.50) to measure convergent validity. Model 2c complies with this standard, and in particular the factor loadings of personal consequences are very high. Only item 5 has a slightly lower factor loading (0.49). The stricter evaluating tool of convergent validity (AVE), however, shows mixed results. Factor 1 with an AVE of 0.537 has a good convergent validity. Factor 2, with an AVE of 0.334, however, has a lower convergent validity. If the AVE is < 0.50, this means that the variance of the measurement error is larger than the variance explained by the factor, which makes the validity of the factor and the individual indicators questionable [48]. The validity of factor 2 is thus less strong than that of factor 1. Nevertheless, all factor loadings are significant and close to or larger than 0.50. Furthermore, the factors have a high discriminant validity, as there are no cross-loadings between the indicators of the two factors and the covariance of the two factors is lower than the threshold of 0.80–0.85.

#### 3.5.2 Concurrent validity

As heavy episodic drinking is linked to negative consequences which students experience [50], we tested whether two drinking variables (binge drinking and AUDIT-c) correlated with Model 2c.

At first, we included AUDIT-c in the model (item 3 as reference category). It appears that the model fit is not as it should be. Although RMSEA and SRMR both have acceptable values (0.059 and 0.051, respectively), CFI and TLI are too low (0.866 and 0.812, respectively). However, since the response to the first question of the AUDIT indicates whether or not the respondents need to proceed with the rest of the AUDIT-questions, the bad model fit could be explained by a possible error term correlation for the first two questions. If the respondents indicated that they had never drunk alcohol before in question 1, they did not need to fill in the whole AUDIT. Consequently, we decided to freely estimate this error term correlation. As a result, the model fit improved substantially (χ^2^ = 459.194; RMSEA = 0.029; SRMR = 0.0426; CFI = 0.968; TLI = 0.954; AIC = 507.194).

**Fig 3:**
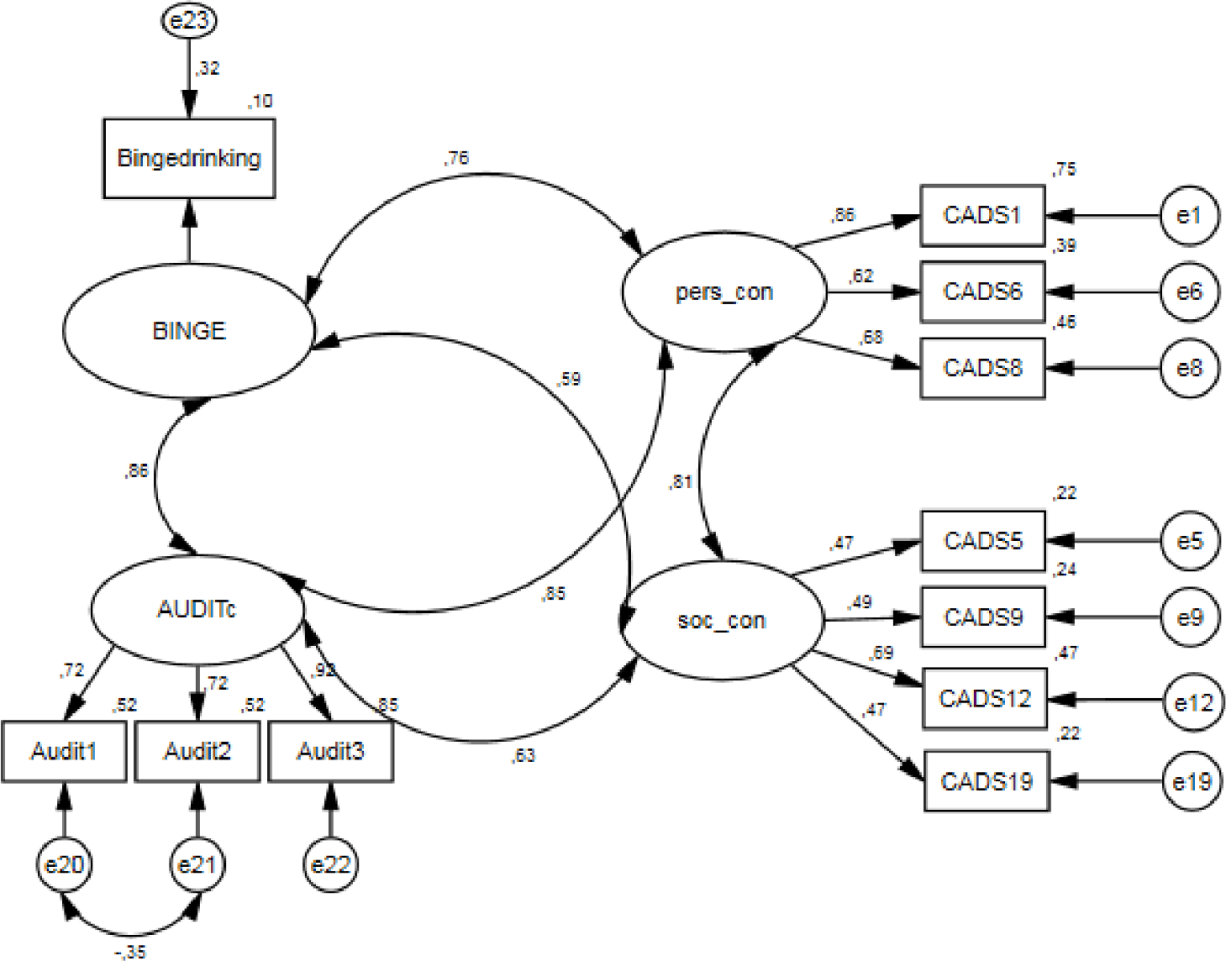
Concurrent validity test of model 2c with variables AUDIT-c and binge drinking.

In a final step we included the variable ‘binge drinking’ as a one-indicator construct (Fig 3). The error variance was equalized to 0.0865 based on the following formula: Var (E) = (1-REL)*VAR (indicator) → Var (E) = (1–0.9) * 0.*865*. An error variance of 0 is not preferred, since misinterpretation of the question is possible. The fit of this final model is very good (χ^2^ = 531.926; RMSEA = 0.029; SRMR = 0.0390; CFI = 0.964; TLI = 0.948; AIC = 587.926). The covariance between AUDIT-c and both personal and social consequences is 0.854 and 0.626, respectively. The covariance between binge drinking and both personal and social consequences is 0.764 and 0.594, respectively. The covariances are significant.

## 4. Discussion

Alcohol research should rely on valid and reliable instruments to measure consequences of alcohol misuse. Although considerable research has used the negative consequences scale of the Core Alcohol and Drug Survey [26–30], little is known about its psychometric properties, especially when not used in English. Therefore, the primary aim of this research was to address the research gap regarding the psychometric properties of a Dutch version of the CADS in a sample of 19,253 Flemish university and college students. We focused especially on alcohol consequences and examined the factor structure of the Dutch CADS by comparing two models from the literature, using confirmatory factor analysis and adapting the models if necessary. Reliability and validity issues were also addressed.

Based on the literature, we started with a one-factor structure containing the 19 items as developed by Presley et al. (1993) and a two-factor structure as suggested by Martens et al. (2005) [24, 31]. These initial models were adapted based on the factor loadings, modification indices and goodness-of-fit indices. As a result, CFA was performed on 6 models and fit indices were compared. In addition, composite reliability was measured for every model. The best model (CADS_D) was a two-factor structure, identifying personal consequences (had a hangover; got nauseated or vomited; missed a class) and social consequences (got into an argument or fight; been criticized by someone I know; done something I later regretted; been hurt or injured) (Model 2c). This model was identified as the best based on both the model fit and composite reliability of the two factors. Although Model 2b had the lowest AIC, and thus the best model fit, the composite reliability of the second factor was not acceptable. Since Model 2c had a much better composite reliability and only a slightly higher AIC, Model 2c was preferred over Model 2b. Our findings confirm the fact that the negative consequences of alcohol misuse should be measured as a two-dimensional scale, focusing not only on consequences that affects the drinkers themselves, but also consequences harming other people around them [23, 31].

Finally, the validity of the CADS_D was assessed. Construct validity was evaluated as acceptable, with good discriminant validity, although the convergent validity of the factor ‘social consequences’ could be improved. Concurrent validity was measured by testing the known correlation of two drinking variables (binge drinking and AUDIT-c) with the negative consequences students experience. Concurrent validity was evaluated as good.

We need to take some limitations of the study into account. Firstly, we excluded the consequences which were encountered by less than 5% of the participants. This does not mean that these consequences were of minor importance. On the contrary, these deleted items are often more severe than the ones included in the analyses and therefore remain important. Secondly, the CADS was measured as an interval variable using frequencies. In this way, a higher weight is given to a student who, for example, experienced a hangover six times last year compared to a student who had been arrested for driving while intoxicated (DWI)/driving under the influence (DUI) twice last year. Future studies should analyze the CADS in a dichotomous way and establish whether the same results are found. And finally, the assessment of the concurrent validity could be improved by measuring the correlation between the CADS and other consequences scales, such as the Young Adult Alcohol Consequences Questionnaire or the Rutgers Alcohol Problem Index [16, 21]. However, these scales were not available in the dataset and thus these analyses could not be performed.

Despite these limitations, the current study aimed to enhance the knowledge of the psychometric properties of the CADS. We did this by addressing the factor structure, reliability and validity of a Dutch version of the CADS in a large sample of 19,253 Flemish university and college students. The study findings have both theoretical and practical implications. Theoretically, the results indicate that a two-factor structure, identifying personal and social consequences, had the best model fit. This current study will help future researchers working with this scale to address alcohol-related consequences correctly. From a practical point of view, the CFA results indicate that the shortened Dutch version of the CADS (CADS_D) is a valid and reliable instrument to screen for alcohol-related consequences among college students, with the ultimate aim of preventing these consequences. Moreover, we expanded the debate about evaluating models and encourage not blindly evaluating model fit, but also taking reliability and validity issues into account.

University and college students: The use of the concepts ‘college’ and ‘university’ differs between countries worldwide. In Belgium, colleges offer professional bachelor degrees, whereas universities offer academic bachelor and master degrees as well as doctoral degrees.

## Supporting information

S1 Table Core alcohol and drug survey–consequences scale. (PDF)

## Abbreviations

ADF: Asymptotic Distribution Free
AIC: Akaike Information Criterion
CADS: Core Alcohol and Drug Survey–in this research the abbreviation refers specifically to the question battery of negative consequences
CFA: Confirmatory factor analysis
CFI: Comparative Fit Index
df: Degrees of Freedom
DUI: Driving under the influence
DWI: Driving while intoxicated
FL: Factor loading
MI: Modification Indices
RMSEA: Root Mean Square Error of Approximation
SRMR: Standardized Root Mean Square Residual
TLI: Tucker-Lewis Index
χ^2^: Chi-square
