# Supplementary material for: The psychometric properties of a shortened Dutch version of the consequences scale used in the Core Alcohol and Drug Survey
Source: PLoS One. 2017 Dec 7;12(12):e0187876. doi: 10.1371/journal.pone.0187876 (PMC5720707; doi:10.1371/journal.pone.0187876)
Supplement: S1 Table — (PDF) [file pone.0187876.s001.pdf]

Item	
**1**	Had a hangover
**2**	Performed poorly on a test or important project
**3**	Been in trouble with police, residence hall, or other college authorities
**4**	Damaged property, pulled fire alarm, etc.
**5**	Got into an argument or fight
**6**	Got nauseated or vomited
**7**	Driven a car while under the influence (DUI)
**8**	Missed a class
**9**	Been criticized by someone I know
**10**	Thought I might have a drinking or other drug problem
**11**	Had a memory loss
**12**	Done something I later regretted
**13**	Been arrested for DWI/DUI
**14**	Have been taken advantage of sexually
**15**	Have taken advantage of another sexually
**16**	Tried unsuccessfully to stop using
**17**	Seriously thought about suicide
**18**	Seriously tried to commit suicide
**19**	Been hurt or injured
